# Menstrual Exile: Nepal’s Chhaupadi and the Policy-Practice Divide

**DOI:** 10.3389/ijph.2024.1608202

**Published:** 2024-12-16

**Authors:** Animesh Ghimire

**Affiliations:** ^1^ Faculty of Medicine, Nursing and Health Science, Monash University, Clayton, VIC, Australia; ^2^ Sustainable Prosperity Initiative Nepal, Kathmandu, Nepal

**Keywords:** Chhaupadi, menstrual exile, Nepal, human rights, menstrual hygiene

## Introduction

Despite criminalization, Chhaupadi, the practice of exiling menstruating women and girls to isolated sheds in Nepal, persists, exposing a stark disconnect between public health policy and reality. This commentary argues that deeply ingrained socio-cultural norms, socioeconomic disparities, and implementation challenges hinder eradication efforts. The practice, linked to severe health risks and human rights violations, underscores a critical need to move beyond legislation and engage communities in dismantling the beliefs that perpetuate menstrual stigma. Empowering girls through education and strengthening law enforcement is crucial. Furthermore, addressing the urban-rural divide by improving access to healthcare, sanitation, and education is vital to creating an enabling environment for change. This commentary calls for a multi-sectoral approach and a united front to achieve a Chhaupadi-free Nepal, where menstruation is no longer a source of shame but a natural and celebrated facet of womanhood.

## Chhaupadi: A Stain on Nepal’s Conscience

The crimson stain of menstruation, a natural biological process, tragically transforms into a mark of shame and exile for countless women and girls in Nepal. The practice of Chhaupadi, where menstruating women and girls are banished to isolated and squalid sheds, exposes a stark and tragic disconnect between public health policy and reality [1]. These “Chhaupadi huts,” ranging from dilapidated stables to basic mud structures, become sites of deprivation and danger [1]. Denied access to basic sanitation, clean water, and adequate shelter, women and girls endure not only physical hardship but also social exclusion [2]. They are rendered untouchable and excluded from participating in communal activities, including attending school while they are menstruating [2].

## The Failure of Policy: A Disconnect Between Law and Life

This enforced isolation into “Chhaupadi huts” carries grave consequences, increasing their vulnerability to infections, hypothermia, and respiratory illnesses, as well as the threat of sexual assault and animal attacks [1]. The recent death of a teenage girl from a snake bite in August 2023 serves as a chilling reminder of the deadly consequences of this persistent practice [3]. Despite being outlawed since 2005 [4], and criminalized in 2017 [5], this harmful tradition continues to inflict physical and psychological harm on countless individuals, particularly in the rural western regions [2]. This commentary argues that the failure to eradicate Chhaupadi exposes a critical disconnect between policy and practice. It highlights the urgent need for a multi-sectoral approach that addresses the deep-seated socio-cultural norms underpinning this discriminatory practice.

## Beyond Legislation: Confronting the Roots of Chhaupadi

While Nepal has made commendable strides in enacting legislation to protect the rights of women and girls [6], the continued prevalence of Chhaupadi underscores the limitations of a top-down approach to public health [7]. Policy enactments, however well-intentioned, are rendered ineffective without robust implementation and community engagement. The current situation begs the question: Who is responsible for this implementation gap? It is tempting to blame government agencies solely for their lack of enforcement. However, Chhaupadi is deeply intertwined with cultural beliefs and religious practices that perpetuate the notion of menstrual impurity [4]. Legal pronouncements or punitive measures do not easily sway these beliefs. In fact, attempts to enforce the ban have often met with resistance from community leaders and traditional healers who wield significant influence within these communities [2].

## A Nation Divided: Bridging the Urban-Rural Chasm

The persistence of Chhaupadi is fueled by the failure of interventions to dismantle the deeply entrenched cultural beliefs and socioeconomic inequalities that sustain it [1]. Despite awareness campaigns, community resistance to change remains strong [2]. Women and girls who defy the practice face ostracization and abuse, perpetuating a cycle of fear and adherence to harmful norms [8]. Furthermore, poverty and inadequate housing in rural communities often make it impossible to accommodate menstruating individuals within the home, forcing them into unsafe sheds [2]. This reality exposes a chasm between urban and rural regions within low- and middle-income countries (LMICs) like Nepal [9]. Ironically, the practice of Chhaupadi has never been prevalent among urban Nepalese women [10], but it remains deeply entrenched in rural regions like Karnali [1], approximately 860 km from the capital city, Kathmandu. This disparity necessitates a shift from a simplistic global north-south dichotomy to a nuanced understanding of the unique challenges within the global south itself.

## Towards a Chhaupadi-Free Nepal: A Call to Action

Eradicating Chhaupadi demands a multi-pronged approach that transcends mere legislation and recognizes the practice as a fundamental human rights violation. Challenging the ingrained cultural beliefs that perpetuate this harmful tradition requires active community engagement, fostering open dialogue, dismantling menstrual stigma, and involving key influencers like religious leaders and traditional healers. Empowering girls through education, particularly comprehensive sexuality education that addresses menstrual hygiene and dispels myths. Furthermore, strengthening law enforcement by allocating adequate resources for monitoring and empowering local authorities is critical to ensure accountability and change. Addressing these socioeconomic disparities between urban and rural areas by improving access to healthcare, sanitation, and education is vital to creating an enabling environment where menstruation is no longer shrouded in stigma and shame. Ultimately, bridging the gap between policy and practice is a moral imperative that demands a united front – government agencies, NGOs, community leaders, healthcare professionals, and individuals alike – to forge a Nepal where menstruation is a natural and celebrated facet of womanhood, not a source of isolation and fear.
